# Long Noncoding RNAs in Response to Hyperosmolarity Stress, but Not Salt Stress, Were Mainly Enriched in the Rice Roots

**DOI:** 10.3390/ijms25116226

**Published:** 2024-06-05

**Authors:** Yanrong Pang, Kaifeng Zheng, Qinyue Min, Yinxing Wang, Xiuhua Xue, Wanjie Li, Heping Zhao, Feng Qiao, Shengcheng Han

**Affiliations:** 1 Beijing Key Laboratory of Gene Resources and Molecular Development, College of Life Sciences, Beijing Normal University, Beijing 100875, China; 202321200016@mail.bnu.edu.cn (Y.P.); kaifeng_zheng@mail.bnu.edu.cn (K.Z.); yinxingwang2024@163.com (Y.W.); xiuhuaxue@bnu.edu.cn (X.X.); lwj@bnu.edu.cn (W.L.); hpzhao@bnu.edu.cn (H.Z.); 2 School of Life Sciences, Qinghai Normal University, Xining 810008, China; m1nq1nyue@163.com; 3 Academy of Plateau Science and Sustainability of the People’s Government of Qinghai Province & Beijing Normal University, Qinghai Normal University, Xining 810008, China

**Keywords:** LncRNAs, PCGs, OSCA1.1, hyperosmolarity stress, salt stress, rice

## Abstract

Due to their immobility and possession of underground parts, plants have evolved various mechanisms to endure and adapt to abiotic stresses such as extreme temperatures, drought, and salinity. However, the contribution of long noncoding RNAs (lncRNAs) to different abiotic stresses and distinct rice seedling parts remains largely uncharacterized beyond the protein-coding gene (PCG) layer. Using transcriptomics and bioinformatics methods, we systematically identified lncRNAs and characterized their expression patterns in the roots and shoots of wild type (WT) and *ososca1.1* (reduced hyperosmolality-induced [Ca^2+^]_i_ increase in rice) seedlings under hyperosmolarity and salt stresses. Here, 2937 candidate lncRNAs were identified in rice seedlings, with intergenic lncRNAs representing the largest category. Although the detectable sequence conservation of lncRNAs was low, we observed that lncRNAs had more orthologs within the *Oryza*. By comparing WT and *ososca1.1*, the transcription level of OsOSCA1.1-related lncRNAs in roots was greatly enhanced in the face of hyperosmolality stress. Regarding regulation mode, the co-expression network revealed connections between *trans*-regulated lncRNAs and their target PCGs related to OsOSCA1.1 and its mediation of hyperosmolality stress sensing. Interestingly, compared to PCGs, the expression of lncRNAs in roots was more sensitive to hyperosmolarity stress than to salt stress. Furthermore, OsOSCA1.1-related hyperosmolarity stress-responsive lncRNAs were enriched in roots, and their potential *cis*-regulated genes were associated with transcriptional regulation and signaling transduction. Not to be ignored, we identified a motif-conserved and hyperosmolarity stress-activated lncRNA gene (*OSlncRNA*), speculating on its origin and evolutionary history in *Oryza*. In summary, we provide a global perspective and a lncRNA resource to understand hyperosmolality stress sensing in rice roots, which helps to decode the complex molecular networks involved in plant sensing and adaptation to stressful environments.

## 1. Introduction

Rice (*Oryza sativa* L.) is one of the most essential food crops worldwide, but its growth is constantly challenged by various adverse abiotic stresses, including cold, heat, nutrient deficiencies, drought, and excess salt or metal toxicity [1,2,3]. Among these, drought and salinity are the primary stressors impacting crop growth and productivity [4]. Osmotic or hyperosmolarity stress, primarily caused by drought, and salt stress, which exerts dual effects on cells including both hyperosmolarity and ion-toxic impacts, are major challenges for rice cultivation [5]. Being sessile, plants have evolved suitable strategies to acclimate to these challenging conditions [6].

Functioning as a second messenger, Ca^2+^ regulates plant growth, development, and reactions to environmental stresses [7,8]. In rice, elevated osmotic pressure and high salinity can initiate early Ca^2+^ signaling, and multi-level responses can encompass the reconstruction of transcriptional networks, transcript processing, translation, post-translational and protein modifications to facilitate adaptation to challenging conditions. The *OSCA* gene (reduced hyperosmolality-induced [Ca^2+^]_i_ increase) has been characterized to code mechanosensitive calcium-permeable channels as osmosensors in plants [9,10,11]. In addition, there are multiple *OSCA* gene copies in the genome, and the *OSCA* gene family has been identified in many species, such as soybean, barley, wheat, and rice [12,13,14,15,16]. In *Arabidopsis*, the OSCA1 protein has been shown as a calcium-permeable channel sensitive to hyperosmotic conditions. Moreover, *OsOSCA1.1* could mediate hyperosmolality-induced [Ca^2+^]_cyt_ increases (OICI_cyt_) and salt stress-induced [Ca^2+^]_cyt_ increases (SICI_cyt_) in rice roots after exposure to hyperosmolality and salt stress [17]. Therefore, exploring the regulatory molecules and mechanisms related to *OSCA* family members is crucial to understanding how plants sense and respond to abiotic stress [3].

Regarding the underlying responses to hyperosmolarity and salt stress in various plants, there have been numerous studies concentrated on the functional analysis of protein-coding genes (PCGs) [18,19,20]. Too many plant noncoding RNAs (ncRNAs) have been recognized as hidden players in development and stress responses [21]. As research on long ncRNAs (lncRNAs) advances, their distinctive biological characteristics in diverse essential processes and responses to environmental stresses have been demonstrated [22]. For instance, the lncRNA *COLDAIR* was crucial for repressing the floral repressor FLOWERING LOCUS C (*FLC*) during vernalization. And the DROUGHT-INDUCED (*DRIR*) lncRNA could regulate responses to abiotic stresses in *Arabidopsis* [23,24]. In rice, various regulation functions of lncRNA, such as in panicle development and fertility, early endosperm development, blast disease resistance, and other aspects, have been validated [25,26,27]. The prerequisite for researching target lncRNAs is the identification and mining of lncRNAs, as has been performed in the published works about various plant groups [28,29,30,31,32,33]. For rice, results on root salt-responsive lncRNAs and leaf drought-related lncRNAs have been reported [34,35]. However, the lncRNA identification and research simultaneously comparing different abiotic stresses and distinct seedling parts is still blank, and there are no clues about the relationship between lncRNAs and *OsOSCA1.1*. In addition, few conserved lncRNAs are related to the hyperosmolality stress response. 

Using transcriptomics and bioinformatics methods, we selected two rice materials (wild type and *ososca1.1*) and conducted systematic lncRNA identification in the roots and shoots of seedlings under abiotic stress. It is worth emphasizing that we compared the different response patterns of lncRNAs in the face of hyperosmolality stress and salt stress. Our results on OsOSCA1.1-related lncRNAs integrated lncRNAs into the regulatory pathways of OSCA family members. Furthermore, the discovery and evolutionary history of the hyperosmolarity stress-activated lncRNA gene in rice (*OSlncRNA*) provides a novel example for understanding the adaptation of rice to hyperosmolarity stress. Combined with *OsOSCA1.1*, this work is a global perspective on lncRNAs in understanding hyperosmolality stress sensing in rice roots. Our results help to decode the complex molecular networks involved in plant sensing and adaptation to stressful environments. 

## 2. Results

### 2.1. A Large Number of Intergenic lncRNAs Exist in Rice Shoots and Roots 

**Figure 1:**
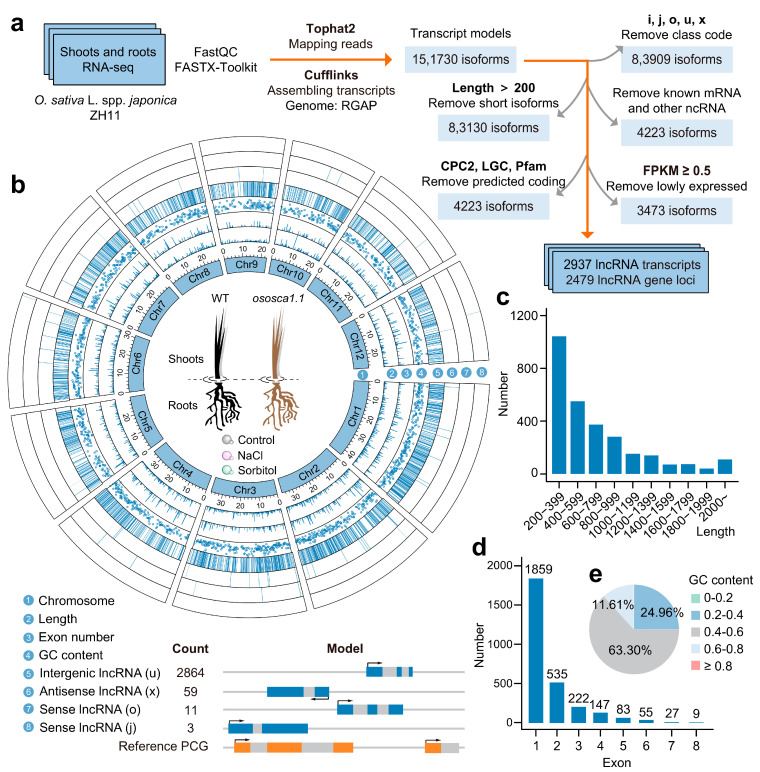
Transcriptome-wide identification and characterization of long noncoding RNAs (lncRNAs) in rice seedlings. (**a**) Bioinformatics workflow about lncRNA identification. In WT and *ososca1.1*, stressed roots and their corresponding shoots were subjected to transcriptome sequencing (n = 12) and lncRNAs scanning, respectively. (**b**) The distribution of lncRNAs among 12 chromosomes and transcriptome-wide characterization of rice seedling lncRNAs (n = 2937). Compared with PCGs, the gene structure pattern diagrams about the four types of lncRNAs point out their differences. (**c**) Length distribution of rice seedling lncRNAs. (**d**) Exon number of rice seedling lncRNAs. (**e**) The proportion of lncRNAs with different GC contents.

To comprehensively pinpoint lncRNAs involved in the processes induced by OsOSCA1.1, salt, and hyperosmolarity stress in rice, we conducted lncRNA identification based on 24 RNA-seq data sets [32]. It contained the seedling roots of ZH11 and *ososca1.1* exposed to the solution (hyperosmolarity stress, salt stress, and control) and their corresponding shoots [17]. After completing the general transcriptome analysis, a total of 151,730 transcript models were eligible to proceed to the subsequent lncRNA screening pipeline. Combining the length, coding potential, expression level, and other properties of transcripts, 2937 isoforms (2479 loci) were considered acceptable lncRNAs (Figure 1 and Table S1). A lncRNA transcriptome-wide profile of rice seedlings depicted the basic characteristics and chromosomal distribution (Figure 1b). LncRNA was widely distributed on the 12 chromosomes of rice, and intergenic lncRNA (lincRNA) was the most dominant type (97.51%) (Figure 1b). Other types of lncRNA were relatively rare, including 59 antisense lncRNAs (lncNATs) and 14 sense lncRNAs (11 transcripts considered as generic exonic overlap with a reference transcript, and three isoforms considered as potential novel transcripts). A series of intergenic non-coding transcripts in rice seedlings have been completely ignored. For the length, most lncRNAs (average length of 729 nt) were comparatively short: 77.83% of transcripts were shorter than 1000 nt, and only 4.09% of lncRNAs exceeded 2000 nucleotides (Figure 1c). Additionally, a significant proportion of lncRNAs were found to have no more than two exons, with fewer lncRNAs containing a higher number of exons (Figure 1d). In terms of GC content, lncRNAs displayed a wide spectrum of variability (from 0.196 to 0.816), with an average value of 0.471. The average GC content of lncRNA was higher than that of the whole rice genome (0.436). This result was consistent with the conclusions of previous lncRNA-related identification work in plants. More than half (63.36%) of the lncRNAs had GC content concentrated between 0.4 and 0.6 (Figure 1e). Therefore, a series of basic statuses of lncRNAs in rice seedlings could also be generally regarded as the characteristics of lincRNAs.

### 2.2. Transcriptional Levels of OsOSCA1.1-Related lncRNAs in Roots Are Greatly Enhanced under Hyperosmolality Stress 

**Figure 2:**
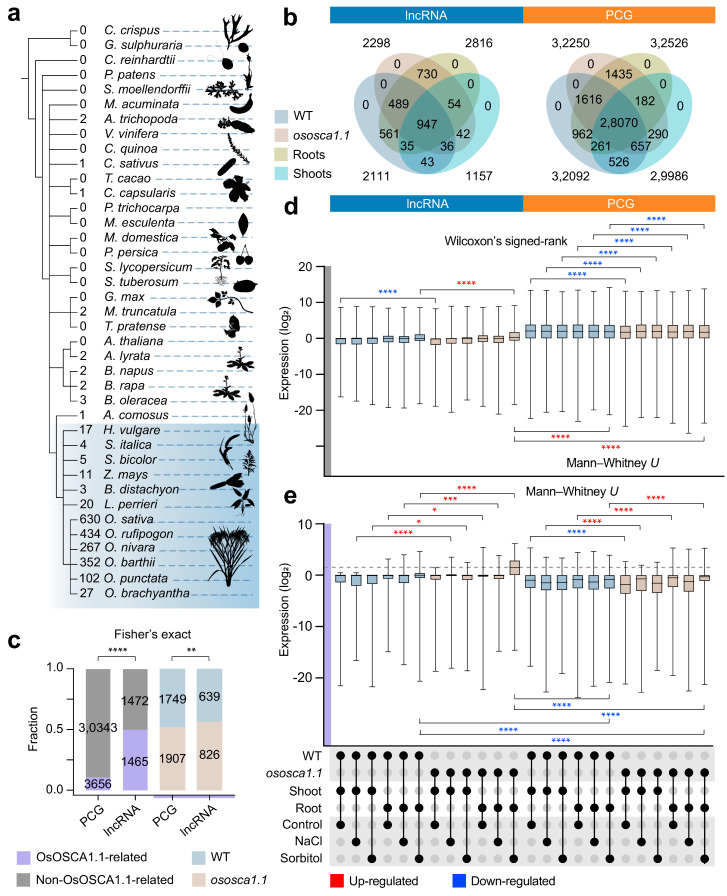
The lncRNA conservation pattern and expression of OsOSCA1.1-related lncRNAs in rice seedlings. (**a**) Phylogenetic distribution of conversed lncRNAs. Sequence conservation of lncRNAs (ZH11 and *ososca1.1*) and other species in CANTATAdb (n = 39, containing *O. sativa*). The counts represent the number of homologs lncRNAs. The images of the different species are from PhyloPic (https://www.phylopic.org/, accessed on 29 April 2024). (**b**) Comparison among members of lncRNAs (n = 2937) and PCGs (n = 33,999) in WT, *ososca1.1*, roots, and shoots. (**c**) Fraction of OsOSCA1.1-related and non-OsOSCA1.1-related lncRNAs and PCGs. (**d**) Expression of non-OsOSCA1.1-related lncRNAs (n = 1472) and PCGs (n = 30,343). (**e**) Expression of OsOSCA1.1-related lncRNAs (n = 1465) and PCGs (n = 3656). Expression levels in each sample are computed in log (FPKM)_2_ units. (*: *p* < 0.05, **: *p* < 0.01, ***: *p* < 0.001, ****: *p* < 0.0001, two-sided Mann–Whitney *U* test, two-sided Fisher’s exact test).

Sequence conservation was regarded as one of the key indicators for understanding molecular and biological functions [36]. To further understand the orthologs of rice seedling lncRNAs among different species, we performed BLAST on lncRNAs from 39 species, containing eudicots, monocots, basal angiosperms, ferns, mosses, and green algae. In monocots, the number of hit lncRNAs was consistent with the known phylogenetic relationships, suggesting that there was a certain degree of conservation in the evolutionary pattern of lncRNAs (Figure 2a and Table S2). Compared with *O. sativa* lncRNAs in the CANTATAdb database, only 630 lncRNAs possessed conserved sequence segments, and 78% of the lncRNAs were newly identified in rice seedlings. Approximately 14.8% of lncRNAs showed detectable sequence homology to lncRNAs in *Oryza rufipogon*, followed by *Oryza nivara*, *O. barthii*, and *O. punctata*. Fewer orthologs in *H. vulgare*, *S. italica*, and *Z. mays* indicated that even in grasses, the sequence conservation of lncRNAs was poor. However, few corresponding transcripts existed in dicotyledonous and basal plant taxa. 

After comparing the number of transcripts in the roots and shoots of WT and *ososca1.1*, we found that the number variation in PCGs between groups was low (Figure 2b, Tables S3 and S4), but that of lncRNAs was the highest in roots and the lowest in shoots. In total, 89% of shoot lncRNAs could be found in roots, but roots only shared 37% of lncRNAs with shoots. Our prior studies showed that *OsOSCA1.1* mediated the perception of hyperosmolality and salt stress, and we tracked the potential OsOSCA1.1-related lncRNAs and PCGs considering the importance of *OSCA* gene family members [15,17]. If the *OsOSCA1.1* retention did not change the presence of transcripts in WT and *ososca1.1* (FPKM ≥ 0.5 in ≥1 sample), then these transcripts were named as non-OsOSCA1.1-related; otherwise, they were OsOSCA1.1-related (Figure 2c). Compared with PCGs, the proportion of OsOSCA1.1-related lncRNAs (approximately 90%) was higher, and only 10% PCGs could be regulated under *OsOSCA1.1* (Figure 2c; ****: *p* < 0.0001, two-sided Fisher’s exact test). Although the number of lncRNAs was relatively small, lncRNAs responded more sensitively to the loss of *OsOSCA1.1* than PCGs (**: *p* < 0.01, two-sided Fisher’s exact test). Because non-regulated transcripts were more reflective of the transcriptional changes brought about by the mutant, we compared the differences in overall transcript levels between WT and *ososca1.1* in non-OsOSCA1.1 regulated lncRNAs and PCGs. The mutation of *OsOSCA1.1* caused a significant decrease in the expression of PCGs, indicating that *OsOSCA1.1* played an important transcriptional role in response to abiotic stress (Figure 2d; ****: *p* < 0.0001, two-sided Wilcoxon’s signed-rank test). For lncRNAs, the expression in the *ososca1.1*-shoot-control group was significantly lower than that of WT (****: *p* < 0.0001, two-sided Wilcoxon’s signed-rank test). Interestingly, the transcript level of the shoot-sorbitol group was significantly increased after the *OsOSCA1.1* mutation (****: *p* < 0.0001, two-sided Wilcoxon’s signed-rank test). Therefore, we proposed that when roots faced hyperosmolality stress, mutation of *OSCA* family members directly activated the lncRNA transcription level in roots. In rice roots, the transcription pattern of OsOSCA1.1-related lncRNAs in response to hyperosmolality stress was different from that of PCGs. At the same time, the low transcription level of lncRNAs was also observed (Figure 2d).

Based on the expression pattern of OsOSCA1.1-related transcripts, we found that the expression of lncRNAs was more affected by *OsOSCA1.1* than that of PCGs. Except for the shoot-control group, the *OsOSCA1.1* mutation did not affect the expression level of OsOSCA1.1-related lncRNAs, and all other groups showed an increase in transcription levels (Figure 2e; *: *p* < 0.05, ***: *p* < 0.001, ****: *p* < 0.0001, two-sided Mann–Whitney *U* test). In the WT root-sorbitol group, the expression of OsOSCA1.1-related lncRNAs was higher than that of PCGs (Figure 2e; ****: *p* < 0.0001, two-sided Mann–Whitney *U* test). However, we did not observe an obvious relationship between salt stress and OsOSCA1.1-related lncRNAs. Therefore, we analyzed the differences in transcription patterns between OsOSCA1.1-related PCGs and lncRNAs. 

### 2.3. Co-Expression Network Points Out the Connection between lncRNAs and Their Trans-Regulated Target PCGs about OsOSCA1.1-Mediated Hyperosmolality Stress Sensing 

**Figure 3:**
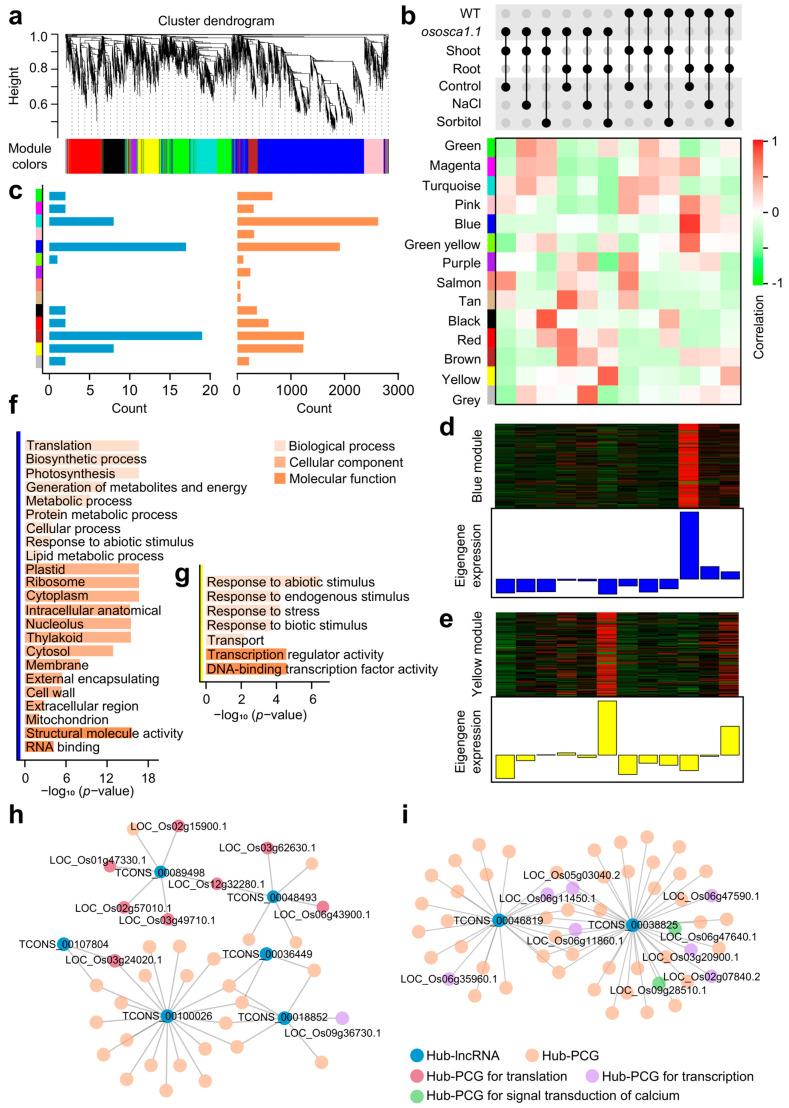
Co-expression network of *trans*-regulated lncRNAs and their PCGs in rice seedlings associated with OsOSCA1.1 and hyperosmolality stress. (**a**) Hierarchical cluster tree and color bands showing the 14 modules by WGCNA (n = 10,000). (**b**) Module–trait correlation analysis. (**c**) Number of lncRNAs and PCGs in each module. (**d**) Eigengene expression profile for the “Blue” module in WT roots (n = 1930). (**e**) Eigengene expression profile for the “Yellow” module in rice roots treated with hyperosmolality stress (n = 1240). (**f**,**g**) Enriched GO terms among PCGs and their *trans*-regulated lncRNAs of the “Blue” module and “Yellow” module. (**h**,**i**) Co-expression *trans*-regulated network of the “Blue” module (weight threshold = 0.4) and “Yellow” module (weight threshold = 0.25). Hub-lncRNAs and Hub-PCGs are represented in blue and orange, respectively, among which PCGs related to translation, transcription, and signaling transduction of calcium functions are highlighted.

To explore the potential functions of *trans*-regulated target PCGs and their lncRNAs in seedlings related to *OsOSCA1.1* and stress, we performed weighted gene co-expression network analysis (WGCNA) on 63 lncRNAs and 9937 PCGs (reserved genes = 10,000) and obtained 14 distinct modules after quality control (Figure 3a,b). The major tree branches in the “Cluster dendrogram” determined the module classification (Figure 3a). “Turquoise”, “Blue”, “Brown”, and “Yellow” were the four bigger modules, and the number of lncRNAs in them was also relatively larger (Figure 3c). Although the count of lncRNAs was small, a series of co-expressed PCGs could provide the possibility of understanding the functions of *trans*-regulated lncRNAs. We were interested in the modules related to the *OsOSCA1.1* gene, and hyperosmolality or salt stress. Therefore, by correlating the modules with samples from different conditions, “Blue” (*p* = 0.00002) and “Yellow” (*p* = 0.0023) modules were found to be significantly associated with the function of *OsOSCA1.1* and the hyperosmolality stress response, respectively (Figure 3b). 

PCGs and lncRNAs in the “Blue” module were highly expressed in roots under natural conditions, and other treatments had less impact on these transcripts (Figure 3d). However, the transcription of genes, that should have been highly expressed in WT roots, almost disappeared in *ososca1.1*. We suspected that this module may be an OsOSCA1.1-mediated “housekeeping module” in rice roots. These PCG transcripts were significantly enriched in “translation”, “biosynthetic process”, “plastid”, “ribosome”, “structural molecule activity”, and “RNA binding” (Figure 3f; *p* < 0.05, Benjamini–Hochberg adjusted, hypergeometric test). They were likely to be downstream regulatory molecules of *OsOSCA1.1* and directly affected the ribosome-related translation process, which in turn led to the expression weakening of the “housekeeping module”. Six *trans*-regulated Hub-lncRNAs were highlighted in the network due to the high eigengene connectivity, and four Hub-lncRNAs (TCONS_00107804, TCONS_00100026, TCONS_00036449, and TCONS_00018852) were related to various metabolic functions and signaling regulation (Figure 3h). Several potential *trans*-regulated target Hub-PCGs, such as dirigent (LOC_Os07g01660.1), leucoanthocyanidin dioxygenase (LOC_Os03g32470.2), auxin-responsive protein (LOC_Os05g48270.1), and cyclin (LOC_Os02g03294.1), could be the fundamental structural regulators in roots. Interestingly, we found that a group of Hub-PCGs connected to TCONS_00089498 and TCONS_00048493 were directly involved in translation activities and owned the ability to bind RNA, such as SWIB/MDM2 domain-containing protein (LOC_Os12g32280.1), RNA recognition motif-containing protein (LOC_Os02g57010.1), and a series of ribosomal proteins such as ribosomal protein L6 (LOC_Os03g24020.1) and ribosomal protein S13p/S18e (LOC_Os03g49710.1) (Figure 3h and Table S5). 

The “Yellow” module possessed the main expression in roots under hyperosmolality stress, and *ososca1.1* directly enhanced the transcription levels of the transcripts in roots (Figure 3e). It indicated that this module was an OsOSCA1.1-mediated hyperosmolality stress-responsive module. The mutation of an *OsOSCA* family member could mobilize the transcription of various other coding and non-coding genes and lead to a stronger response to hyperosmolality stress. These PCGs were significantly associated with “response to abiotic stimulus”, “response to endogenous stimulus”, “transcription regulator activity”, and “DNA-binding transcription factor activity” (Figure 3g; *p* < 0.05, Benjamini-Hochberg adjusted, hypergeometric test). Moreover, we identified two *trans*-regulated Hub-lncRNAs (TCONS_00046819 and TCONS_00038825), which were related to transcriptional regulatory activities, such as AP2 domain-containing protein (LOC_Os06g47590.1) and MYB transcription factor (LOC_Os03g20900.1) (Figure 3i). Unexpectedly, we also found potential *trans*-regulated target PCGs involved in calcium signaling transduction: EF hand family protein (LOC_Os09g28510.1) and calmodulin-related calcium sensor protein (OsCML29, LOC_Os06g47640.1) (Figure 3i). 

### 2.4. Compared with PCGs, lncRNAs Are More Sensitive to Hyperosmolarity Stress Than to Salt Stress in Rice Roots

**Figure 4:**
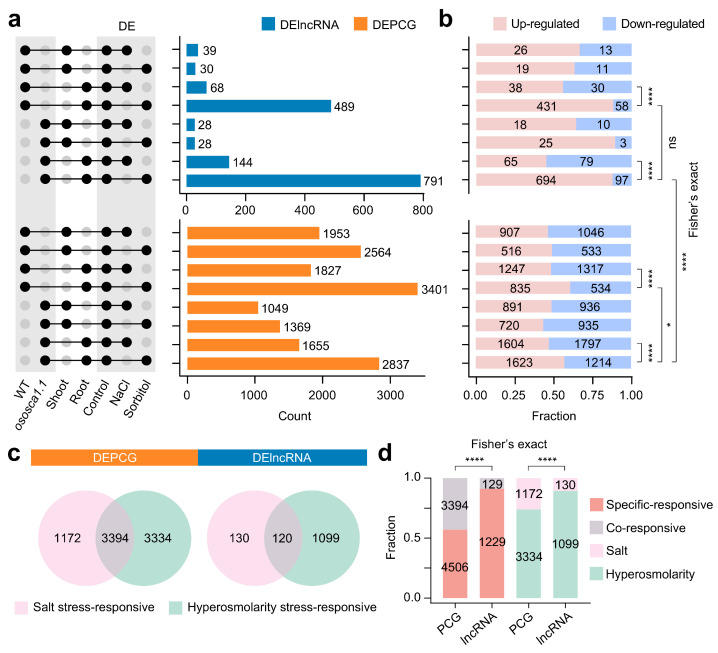
Comparison of different stress-responsive lncRNAs and PCGs. (**a**) Number of DElncRNAs and DEPCGs in response to NaCl or sorbitol in the shoots and roots. (**b**) Fraction of up-regulated and down-regulated DElncRNAs and DEPCGs in rice seedlings. (**c**) Co-responsive (both salt and hyperosmolarity stress-responsive) DElncRNAs and DEPCGs. (**d**) Fraction of salt stress-responsive and hyperosmolarity stress-responsive lncRNAs and PCGs. (*: *p* < 0.05, ****: *p* < 0.0001, two-sided Fisher’s exact test).

To understand the possible roles of lncRNAs in response to salt and hyperosmolarity stress, we carried out differential expression analysis on each sample subjected to NaCl or sorbitol treatment in comparison to the corresponding control. We identified significant differentially expressed lncRNAs (DElncRNAs) and protein-coding genes (DEPCGs) according to the same standard: |log_2_ (fold change)|values ≥ 0.5, *p*-value ≤ 0.05, and *q* value ≤ 0.05 (Figure 4a and Tables S6 and S7). In WT, we characterized 39 and 30 shoots as DElncRNAs and 68 and 489 roots as DElncRNAs in response to NaCl or sorbitol, respectively (Figure 4a). In *ososca1.1*, there were 28 and 28 DElncRNAs in the shoots and 144 and 791 DElncRNAs in the roots in response to NaCl or sorbitol, respectively. After the roots of *ososca1.1* were subjected to hyperosmolarity stress, there were more DElncRNAs. In addition, it was observed that DElncRNAs were mainly up-regulated, which was significantly different from the distribution pattern of DEPCGs (Figure 4b). In roots, hyperosmolarity stress significantly initiated more up-regulated DElncRNAs than salt stress, and this state was not dependent on *OsOSCA1.1* (*: *p* < 0.05, ****: *p* < 0.0001, two-sided Fisher’s exact test). Compared with PCGs, lncRNAs owned different transcriptional response patterns under salt stress and hyperosmolarity stress. Furthermore, 1099 lncRNAs and 3334 PCGs were hyperosmolarity stress-responsive transcripts specifically, whereas 130 lncRNAs and 1172 PCGs were exclusively responsive to salt stress (Figure 3c). LncRNAs in roots were more responsive to hyperosmolarity stress than salt stress, both in terms of number and proportion (Figure 3d; ****: *p* < 0.0001, two-sided Fisher’s exact test). We identified transcripts that responded to both stresses as co-responsive, and the proportion of specific-responsive lncRNAs was higher compared to PCG (Figure 3d; ****: *p* < 0.0001, two-sided Fisher’s exact test). These data once again emphasized that PCGs and lncRNAs had different transcriptional responses to abiotic stress, indicating that lncRNAs in rice roots could be a unique angle to analyze the difference between salt stress and hyperosmolarity stress. 

### 2.5. OsOSCA1.1-Related Hyperosmolarity Stress-Responsive lncRNAs Are Enriched in Roots, and Their Potential cis-Regulated Genes Are Closely Related to Transcriptional Regulation and Signaling Transduction 

**Figure 5:**
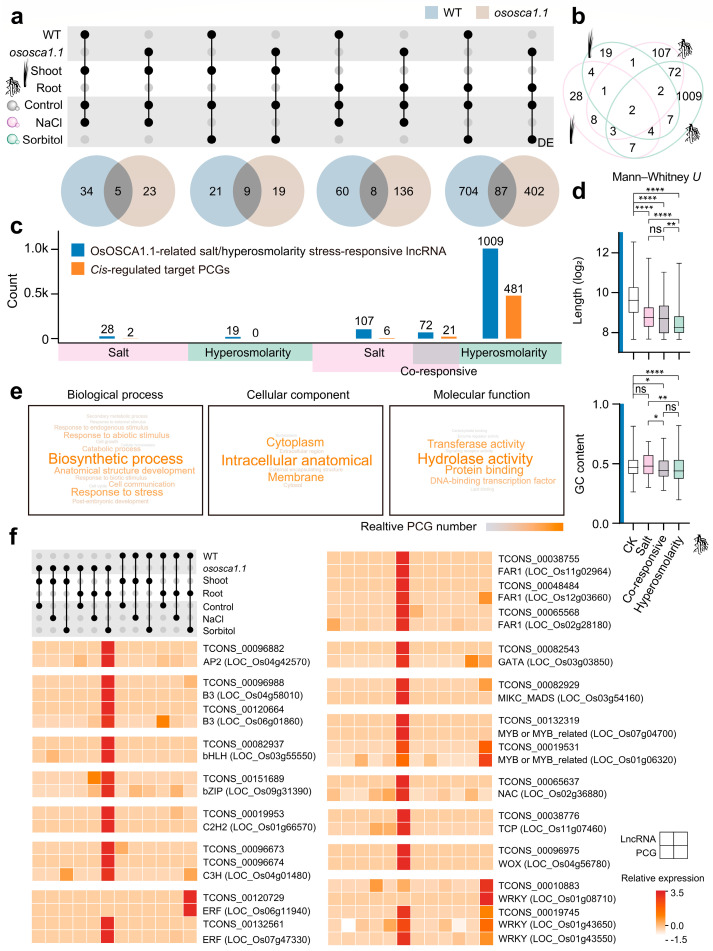
Functions of OsOSCA1.1-related salt/hyperosmolarity stress-responsive lncRNA. (**a**) Selection of OsOSCA1.1-related salt/hyperosmolarity stress-responsive lncRNAs in rice shoots and roots. (**b**) Identification of OsOSCA1.1-related salt/hyperosmolarity stress-responsive lncRNAs specific to shoots and roots. (**c**) Number and comparison of *cis*-regulated target genes and their OsOSCA1.1-related stress-responsive lncRNAs. (**d**) Length and GC content of OsOSCA1.1-related stress-responsive lncRNAs. CK group is non-OsOSCA1.1-related stress-responsive lncRNAs (n = 1663). (**e**) Biological process, cellular component, and molecular function of OsOSCA1.1-related hyperosmolarity stress-responsive *cis*-regulated target PCGs. The color depth and size of the text represent the relative number of genes with related functions. (**f**) Expression pattern of *cis*-regulated target TFs of lncRNAs in rice roots. (Log scale: base = 2.0; log width = 1.0; row scale: normalized). (ns: *p* > 0.05, *: *p* < 0.05, **: *p* < 0.01, ****: *p* < 0.0001, two-sided Mann–Whitney *U* test).

In addition to *trans*-regulation, lncRNAs have also been found to modulate PCGs located nearby in the genome, that is, *cis*-regulation [37]. To explore the lncRNAs related to OsOSCA1.1 about stress sensing, we identified 57 OsOSCA1.1-related salt stress-responsive lncRNAs and 40 OsOSCA1.1-related hyperosmolarity stress-responsive lncRNAs in shoots (Figure 5a). In roots, there were 196 OsOSCA1.1-related salt stress-responsive lncRNAs and 1106 OsOSCA1.1-related hyperosmolarity stress-responsive lncRNAs. Among them, we only cared about specific OsOSCA1.1-related stress-responsive lncRNAs in the four groups of different tissues and treatments (Figure 5b,c). We reported the characteristic differences between OsOSCA1.1-related salt stress-responsive lncRNAs and hyperosmolarity stress-responsive lncRNAs. The number of OsOSCA1.1-related hyperosmolarity stress-responsive lncRNAs was about 10 times that of salt stress-responsive lncRNAs. In addition, the transcript length was significantly smaller, and the GC content was lower compared to salt stress-responsive lncRNAs (Figure 5d; *: *p* < 0.05, ***: *p* < 0.001, ****: *p* < 0.0001, two-sided Mann–Whitney *U* test). It suggested that OsOSCA1.1-related lncRNA had a different responsive structural basis when facing salt and hyperosmolarity stress. 

Furthermore, Bedtools was utilized to identify potential *cis*-regulated target PCGs located within 100 kb upstream and downstream of lncRNAs. Through Pearson correlation coefficient analysis (PCC ≥ 0.9), a series of *cis*-regulated target PCGs were scanned. Figure 5c compared the target genes of lncRNAs, and there were 481 *cis*-regulated target PCGs of OsOSCA1.1-related hyperosmolarity stress-responsive lncRNAs in roots, while the number of salt stress-related target genes in roots was very small (Table S8). In addition, co-responsive lncRNAs might play a role in sensing both salt and hyperosmolarity stress, and 21 potential PCGs had a high expression correlation with 72 lncRNAs. The length and GC content of co-responsive lncRNAs showed a transition state between salt stress-responsive and hyperosmolarity stress-responsive lncRNAs (Figure 5c,d; ns: *p* > 0.05; *: *p* < 0.05, ***: *p* < 0.001, ****: *p* < 0.0001, two-sided Mann–Whitney *U* test). To further understand the related functions of this characteristic component, we organized GO entries (level 3) for the genes. *Cis*-regulated target PCGs of OsOSCA1.1-related hyperosmolarity stress-responsive lncRNAs were mainly associated with “biosynthetic process”, “response to stress”, “cytoplasm”, “intracellular anatomical structure”, “hydrolase activity”, and “protein binding” (Figure 5e). 

In molecular function terms, “DNA-binding transcription factor” triggered the interest in examining TFs because the role of TFs in plant abiotic stress responses could not be ignored. We conducted TF screening and found a total of 26 TFs (15 families), which were documented to participate in stress adaptation (Figure 5f). APETALA2 (AP2), B3, basic helix–loop–helix (bHLH), basic leucine zipper (bZIP), C2H2, cysteine 3 histidine (C3H), ethylene response factor (ERF), far-red-impaired response 1 (FAR1), GATA, MIKC-type MADS (MIKC_MADS), v-myb avian myeloblastosis viral oncogene homolog (MYB or MYB-related), NAM, ATAF1/2 and CUC2 (NAC), teosinte branched1/cincinnata/proliferating cell factor (TCP), WUSCHEL-related homeobox (WOX) and WRKY TF family had members as potential *cis*-regulated target PCGs of lncRNAs. In the “cell communication”, many protein kinases (such as receptor-like protein kinase (LRR), ethylene receptor (LOC_Os02g57530), and coronatine-insensitive protein (LOC_Os03g15880)) that were closely related to stress signaling transduction were identified as potential *cis*-regulated targets of lncRNAs (Figure S2). Most of the gene pairs had similar expression patterns with the “Yellow” module, suggesting that the deletion of *OsOSCA1.1* induced a wide range of *cis*-regulated TFs and signaling transduction to respond to root hyperosmolarity stress. 

### 2.6. An Oryza-Specific Hyperosmolarity Stress-Activated lncRNA Possesses Conserved Motif Architecture 

**Figure 6:**
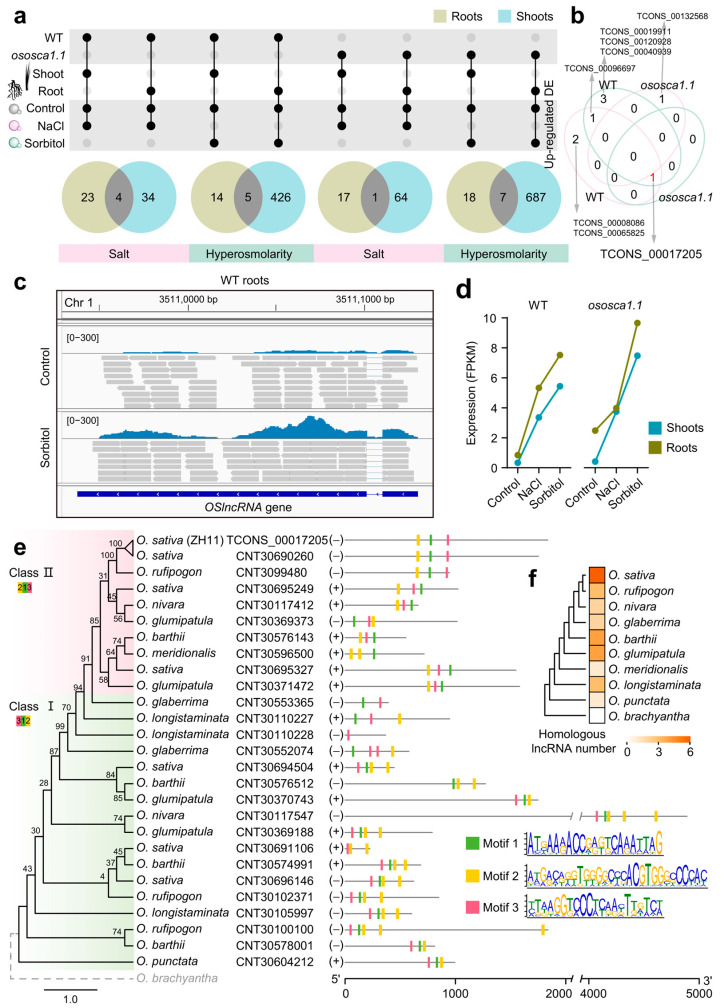
Screening and conservation of stress-activated lncRNAs in rice. (**a**) Selection of up-regulated DElncRNAs both in rice shoots and roots (stress-activated lncRNAs). (**b**) Common and unique stress-activated lncRNAs. (**c**) Genomic location and transcription peak of TCONS_00017205 (*OSlncRNA*) in WT roots using IGV. (**d**) *OSlncRNA* expression patterns in aboveground and underground parts under different stress treatments. (**e**) Phylogenetic tree and motif evolution history of *OSlncRNA* and its homology (Bootstrap = 5000). Relative transcription directions are indicated by positive (+) and negative signs (−). (**f**) Homologous lncRNA numbers in ten *Oryza* species.

Here, we explored the characteristics of aboveground lncRNAs after the belowground parts were stressed. Comparing the shared transcripts, almost all shoot lncRNAs were included in the lncRNA collection of roots, suggesting that the number distribution patterns of seedling lncRNAs in the aboveground and underground parts were different (Figure 2b). Moreover, lncRNAs possessed higher expression levels in roots (Figure 2d and Figure S3a; ****: *p* < 0.0001, two-sided Wilcoxon’s signed-rank test). Using average expression value as the overall transcript level, we found hyperosmolarity stress could better characterize the difference in response patterns between shoots and roots than salt stress (Figure S3b). After the *OsOSCA1.1* mutation, hyperosmolarity treatment stimulated the transcription level of lncRNAs in roots, but PCGs did not appear in this state (Figure S3b). After abiotic stress treatment of roots, some transcripts in both aboveground and underground parts were significantly differentially up-regulated, and we regarded them as stress-activated lncRNAs in seedlings (Figure 6a). There were two salt stress-activated lncRNAs and three hyperosmolarity stress-activated lncRNAs in WT. Interestingly, TCONS_00017205 (from XLOC_006065 gene locus) was salt and hyperosmolarity stress-activated in WT but was only hyperosmolarity stress-activated in *ososca1.1* (Figure 6b). XLOC_006065 was located on chromosome 1 (Chr 1: base pairs 35,111,306 to 35,109,378) (Figure 6c). Transcript levels in underground parts were higher than in aboveground parts, and both stress treatments (especially sorbitol) could stimulate the expression of TCONS_00017205 (Figure 6d). Therefore, we named the gene locus of TCONS_00017205 as the *OSlncRNA* (hyperosmolarity stress-activate lncRNA) in rice. Examining its neighbor PCGs and screening according to the same stress-activated rule, there were two potential *cis*-regulated target genes coding WRKY108 (LOC_Os01g60600.1) and serine/threonine-protein kinase (NAK, LOC_Os01g60700.1) (Figure S3c). After *ososca1.1* was subjected to hyperosmolarity stress, the transcription level of *OSlncRNA* and its *cis*-regulated PCGs could be enhanced in the shoots. 

In fact, TCONS_00017205 was CNT30690260 in the CANTATAdb database because the genome position and sequence of the two are almost identical (score = 1867, E-value = 0). With the help of ten published lncRNA sequence sets in *Oryza*, we tracked the homologous lncRNAs of TCONS_00017205 (Table S9). After the ancestral sequence (CNT30604212) first appeared in *O. punctata*, the homologous members continued to expand in various *Oryza* species (Figure 6e). Combined with the phylogenetic tree, the lncRNA family could be divided into two main clusters (class 1 and 2). Almost all lncRNA family members had three conserved motifs (motif 1, 2, and 3), and these motifs had already appeared in *O. punctata*. The lncRNAs from class 1 were older original lncRNAs with “312” motif arrangement order (5′-3′), while the lncRNAs in class 2 owned the “213” motif architecture. We noticed that motif 2 always showed expansion events in different members, and the lncRNA transcription exit directions were different. The number of conserved lncRNAs in drought-tolerant wild rice (such as *O. barthii*, *O. glimipatula*, and *O. longistaminata*) was relatively high, while there were six paralogous lncRNAs in the cultivated rice *O. sativa*, suggesting that these homologous lncRNAs might mediate drought stress response and adaptation (Figure 6f). In summary, *OSlncRNA* was a young lncRNA, but its conversed motifs had an early evolutionary origin. This lncRNA family might be a conserved functional family that was activated by hyperosmolarity stress. 

## 3. Discussion

LncRNAs have been dismissed as transcriptional noise in the genome for years [38]. With the development of sequencing technology and transcriptome analysis, an increasing number of potential roles of lncRNAs have been unveiled [39,40,41]. A few studies have confirmed the critical involvement of lncRNAs in key biological processes and validated their integral role in the functioning of rice [26,40]. In previous research, lncRNAs exhibited low detectable sequence conservation across diverse plant species [42,43,44]. The limited conservation levels could be inherent to the fast-evolving nature of lncRNAs, establishing a species-specific regulatory stratum in which lncRNAs modulate gene expression through diverse mechanisms [45]. As we observed in *Oryza*, lncRNAs have detectable sequence conservation at the genus level (Figure 2a and Figure 6e). 

### 3.1. LncRNAs of cis- and trans-Regulation Enrich Regulatory Networks of Abiotic Stress 

Since the discovery of the *OSCA* family, its members have been identified and characterized in many species [12,13,15,16]. The protein structure and biological function of *OSCA1* have been reported because it plays an important role in plant sensing of hyperosmolality stress [9,10,11,17]. Because the relationship between lncRNAs and *OsOSCA1.1* had not been reported, we specifically used the *ososca1.1* material to explore OsOSCA1.1-related lncRNAs (Figure 2). The mutation of *OsOSCA1.1* could significantly perturb the transcription level of lncRNAs, which was different from PCGs. Interestingly, in the face of hyperosmolality stress, highly expressed lncRNAs were stimulated in the roots of *ososca1.1* and we wanted to know the involved PCGs and biological significance. Regarding the regulatory mode of lncRNAs, it is mainly divided into *cis*- and *trans*-regulation, and a series of studies have reported the regulatory mode of lncRNA in plants [21,25]. With the help of the co-expression network, we identified two interesting modules and the *trans*-regulated Hub-lncRNAs and their target PCGs. The “Yellow” module was mainly expressed in roots under hyperosmolality stress, and mutation of *OsOSCA1.1* directly enhanced the transcription levels of several Hub-lncRNAs and Hub-PCGs. Therefore, the *OsOSCA1.1* mutation may enhance the transcription of lncRNAs and many TFs, thereby improving the function of the OsCML29 protein and causing a compensatory response to hyperosmolality stress [46,47,48]. In fact, there are very few reports on calcium sensors and signaling transduction in plant lncRNAs. As intracellular Ca^2+^ sensors, the most critical molecules are Ca^2+^-binding proteins (CBPs), including calmodulin (CaM), CaM-like proteins (CMLs), calcineurin B-like proteins (CBLs), etc. [49]. One or more E–F hands are their most important domains, and the “Yellow” module happens to contain two potential CBPs and *trans*-regulated lncRNAs. These co-expressed Hub-lncRNAs and other Hub-PCGs are likely to be potential regulators of a biologically active Ca^2+^/CBP complex. In addition, there was a group of Hub-lncRNAs that appeared to be intertwined with ribosome-related translation processes and were expressed only in WT roots [50,51]. In previous biological pathways and molecular functional annotation of DEPCGs participating in osmotic stress and salt response in *Z.xanthoxylum* roots, the ribosome was identified as significantly enriched [52]. Because the *ososca1.1* plant was extremely sensitive to drought stress, we highly suspected that lncRNAs could interact with *OsOSCA1.1* through *trans*-regulation as a result of maintaining the normal translation of basic root genes. We need to admit that the identity of *OSOSCA1.1* in the above regulatory network is complicated. However, the OsOSCA1.1-related lncRNAs we recorded for the first time and the *trans*-regulated lncRNAs can provide new insights into *OSCA* gene function about sensing hyperosmolality stress. 

Globally, drought stress-related events caused by erratic rainfall patterns always impact plant growth in hyperosmotic environments. In addition to hyperosmotic stress, plants growing in saline-alkali soils also suffer from Na-induced ion stress. To adapt to abiotic stresses, plants possess diverse physiological, biochemical, and molecular mechanisms, including the modulation of various coding and non-coding genes, and cascades of signaling transduction pathways [3,53]. Recent studies have also used co-expression networks and other strategies to demonstrate that lncRNAs were extensively involved in plant responses to drought stress [53,54,55,56]. In crops, salt-stress-related lncRNAs are also the focus of attention [57,58,59]. In our study, lncRNAs showed tissue specificity with a significantly larger number of differentially expressed lncRNAs in roots than in shoots, indicating that the lncRNAs responsive to salt and hyperosmolarity stress were primarily enriched in roots. Interestingly, similar response patterns in response to drought and salt stress in aboveground and underground parts of cotton and tobacco were reported in previous studies [60,61]. After mining salt stress-responsive and hyperosmolarity stress-responsive transcripts, we found that root lncRNAs were insensitive to salt stress, which was completely different from PCGs (Figure 4). Here, we provide an opportunity to understand the differences in salt and hyperosmolarity stress perception in rice seedlings from the perspective of lncRNAs. More importantly, through *cis*-regulation, we found a large number of OsOSCA1.1-related hyperosmolarity stress-responsive lncRNAs in roots but not salt stress-responsive lncRNAs. Further comparison of the length and GC content of these two groups of lncRNAs revealed that hyperosmolarity stress-responsive lncRNAs had lower characteristics. Shorter lncRNAs may require only a short transcription time to respond massively and rapidly to hyperosmolarity stress, and lncRNAs with low GC content were likely to be the product of adaptation evolution to stressful environments [62]. In addition, there were abundant genes encoding TFs around the genome loci of OsOSCA1.1-related hyperosmolarity stress-responsive lncRNAs. Too many studies have proven the necessity of the modulatory relationship between TFs (such as MYB, bZIP, WRKY) and lncRNAs when plants face drought or salt environments, so we can easily understand the intertwining of *OsOSCA1.1* with lncRNAs and TFs [23,63,64,65,66]. Protein phosphylation/dephosphorylation are major signaling events induced by osmotic stress in higher plants. According to previous studies on lncRNAs related to stress, we also discovered the connection between lncRNAs and a series of kinases including serine/threonine protein kinase [67,68]. Especially, the function of serine/threonine protein kinase TaSnRK2.4 involved in the regulation of enhanced osmotic potential, growth, and development under both normal and stress conditions in Arabidopsis was characterized [69]. As a molecule that senses hyperosmolarity stress in roots, the regulatory network of *OsOSCA1.1* must involve complex signaling transduction and transcriptional regulation [64]. If the emergence of hyperosmolarity stress-responsive lncRNAs might regulate the expression of kinase and TFs, it provides a perspective for understanding the importance of *OsOSCA1.1*. On the one hand, plants employ a variety of different adaptive mechanisms to ensure their survival and growth when facing different types of stress, and the hyperosmolarity stress-induced signaling pathway may rely more on the regulation of lncRNA in the roots of rice. On the other hand, the impact of hyperosmolarity stress induced by sorbitol treatment may outweigh the impact of ion toxicity induced by NaCl. Therefore, more lncRNAs were expressed to cope with hyperosmolarity stress to maintain normal rice growth and development. In the genomic, transcriptional, post-transcriptional, and epigenetic levels, analyzing the specific regulation of lncRNAs had significant challenges and difficulties [26]. In *Medicago truncatula*, the response pattern of lncRNAs involved in hyperosmolarity and salt stress was different from rice, which could be determined by the species specificity of Asian cultivated rice [70,71]. Although we cannot explain why hyperosmolarity stress induced more lncRNAs than salt stress, we suspected that this was due to the species specificity and was characterized by the short length and low GC content of lncRNAs. 

### 3.2. Evolutionary History of a Conserved and Hyperosmolarity Stress-Activated lncRNA Gene 

Due to ignoring lncRNAs as an important regulator, our previous studies based on PCGs did not find differences in transcripts with different identities in shoots and roots, whether in number distribution pattern, stress-response pattern, or expression pattern [17]. By systematically comparing aboveground and underground parts of seedlings, we reported a conserved lncRNA gene (*OSlncRNA*) for the first time, which was hyperosmolarity stress-activated (Figure 6). Since the sequence conservation of lncRNAs is weak, it is important to explain and track the evolutionary origin and history of *OSlncRNA* [72]. During the second of two rapid radiation events that occurred in the *Oryza* genus, a gene containing three consecutive motifs (the “312” pattern) appeared in *O. punctata*, a wild rice species native to Africa. With the evolution of *Oryza* and adaptation to different environments, a series of wild rice and cultivated rice species emerged successively, and the locus of this lncRNA gene in the genome continued to expand (Figure 6f). In fact, wild relatives such as *O. rufipogon*, *O. longistaminata*, and *O. glumaepatula*, which can improve the drought tolerance of cultivated rice, possess a series of molecules related to stress signal perception and response in their genomes and transcriptomes [73]. The orthologous lncRNAs containing the “312” motif pattern also had a relatively high copy number in these species, suggesting that this lncRNA gene family is closely related to hyperosmolarity stress. Furthermore, due to gene duplication events (tandem, tetraploid, segmental, trans-positional) during species genome evolution, lncRNAs with a new “213” motif architecture had the opportunity to appear [74,75]. Importantly, “213” pattern lncRNAs had the highest number of paralogs in *O. sativa*, suggesting that this lncRNA family could become a key gene family in the process of rice domestication [76,77,78]. Therefore, we found that the young *OSlncRNA* gene had an obvious transcriptional feature mediated by hyperosmolarity stress and was the NAT lncRNA gene [79]. The “312” or “213” motif architecture is the potentially conservative combination of functional motifs or even modular structural domains [80,81,82,83]. Taxon-specific coding and non-coding RNA genes have been found to possess a profound impact on lineage-specific phenotypic diversification and adaptation [36,84]. The identification of *Oryza*-specific lncRNAs provides a novel example for understanding the adaptation of rice to hyperosmolarity stress. 

### 3.3. Concluding Remarks and Future of Plant lncRNA Biology 

In summary, we are the first to discuss the results on OsOSCA1.1-related lncRNAs, which compiled lncRNAs into the regulatory pathways of *OSCA* family members. We conducted a global investigation of the *cis*- and *trans*-regulatory patterns of lncRNAs in rice seedlings from the transcriptome level and provided help in understanding the evolution and function of plant lncRNAs [22]. Based on short reads RNA-seq, transcript models of lncRNA genes can be identified through the transcript start site (TSS), alternative splicing (AS) site and polyadenylation site of the transcript. However, building transcript models only from short reads is challenging due to the limited length of each read, and the sequence redundancy of the genome [85]. Trimethylation of lysine 4 and lysine 36 in histone H3 (H3K4me3 and H3K36me3 marks), tiling arrays, cap analysis of gene expression (CAGE), 3P-seq and other methods combined with RNA-seq can further reliably identify multi- and single-exon lncRNAs [80]. Although the study of lncRNAs is limited by their extremely poor sequence conservation, the interactions of lncRNAs with cellular factors, namely proteins, DNA, and other RNA molecules, have been demonstrated from a series of levels: pretranscriptional (*APOLO*, *AUXIN REGULATED PROMOTER LOOP*), transcriptional (*Sep3*, *SEPALLATA3*), post-transcriptional (*IPS1*, *induced by phosphate starvation 1*) [85]. More multi-omics studies, comparative genomic and comparative transcriptomic studies are needed to comprehensively analyze the functions of lncRNAs in response to hyperosmolarity or salt stress. Exploring the hyperosmotic response mechanism and evolution of lncRNAs in different plants is a new perspective for understanding abiotic stress. Of course, the experimental verification of taxon-specific lncRNAs can further provide a new understanding of plant responses to abiotic stress, especially hyperosmolarity stress. 

## 4. Materials and Methods

### 4.1. Plant Materials

The rice (*Oryza sativa* L. spp. *japonica* cv. Zhonghua11, ZH11) was planted in the greenhouse and paddy fields of Beijing Normal University, Beijing, China. A T-DNA insertion line of rice was obtained from Huazhong Agricultural University, and we named it *ososca1.1* (genetic background ZH11) [86]. The seedlings at the four-leaf stage (about 30 days old) were treated in Yoshida’s culture solution containing either 250 mM sorbitol or 125 mM NaCl for 24 h. Simultaneously, seedlings were collected after growing in the solution without sorbitol or NaCl as natural growth control. Additional details on seedling materials (shoots and roots) and transcriptome sequencing information can be found in the published paper [17]. The 24 paired-end RNA-seq data sets were downloaded from the National Center for Biotechnology Information (NCBI) Sequence Read Archive (Accession: PRJNA544967).

### 4.2. Transcriptome Assembly and lncRNA Identification 

To ensure high-quality data for subsequent analysis steps, FastQC (version 0.12.1; http://www.bioinformatics.babraham.ac.uk/projects/download.html#fastqc, accessed on 20 September 2023; -q 20 -p 90) and FASTX-Toolkit (version 0.0.14; hannonlab.cshl.edu/fastx toolkit/, accessed on 20 September 2023; -f 7) were utilized to control the quality of the raw reads. The rice reference genome and annotation file were retrieved in the Rice Genome Annotation Project (RGAP, http://rice.uga.edu/, accessed on 20 September 2023) [87]. We employed Bowtie2 (version 2.3.5.1; http://sourceforge.net/projects/bowtie-bio/files/bowtie2, accessed on 20 September 2023) to build the genome index. The clean reads of each sample were mapped to the reference genome using TopHat2 (version 2.1.1; https://ccb.jhu.edu/software/tophat/index.shtml, accessed on 20 September 2023; -I 5000). Furthermore, Cufflinks (version 2.2.1; http://cole-trapnell-lab.github.io/cufflinks/install/, accessed on 20 September 2023) were utilized for transcriptome assembly and quantifying expression [88]. The fragments per kilobase of transcript per million mapped reads (FPKM) values for each transcript were computed using the Cuffdiff program in the Cufflinks package (Table S3).

To make the transcript fit the lncRNA characteristics, we employed an established pipeline to identify lncRNAs in the rice shoots and roots (Figure 1a) [32]. The transcript models were mainly compared with PCG transcripts and classified. We removed other class code types and kept the following five kinds: transcripts falling entirely within a reference intron (class code “i”), generic exonic overlap with a reference transcript (class code “o”), intergenic transcripts (class code “u”), potentially novel isoforms (class code “j”), and natural antisense transcript (class code “x”) (http://cole-trapnell-lab.github.io/cufflinks/cuffcompare/, accessed on 20 September 2023). Transcripts with a nucleotide (nt) length greater than 200 were retained, as this was one of the basic definitions of lncRNAs. Known mRNAs and other non-coding RNAs (tRNAs, rRNAs, snRNAs, and snoRNAs) were removed based on the database (https://plants.ensembl.org/info/data/ftp/index.html, accessed on 25 October 2023) using BLAST (E-value < 10^−10^, identity > 90%). Then, the coding potential for the remaining transcripts was assessed by CPC2, LGC, and Pfam-scan (version 1.6.4, E-value < 10^−5^) [89,90,91]. Only transcripts without protein-coding potential predicted by all the above three software were regarded as candidate lncRNAs. As some lncRNAs could produce miRNAs, we performed BLAST using miRNAs in the rice genome and mature miRNA sequences from miRbase (E-value < 10^−10^, identity > 90%) [92]. Finally, considering that lncRNAs were characterized by low transcription levels, we removed isoforms whose FPKM value was not greater than or equal to 0.5 in any sample set. Only transcripts that passed the above screening pipeline were considered the final acceptable lncRNAs. 

### 4.3. Basic Characteristics and Differential Expression Analysis of Transcripts 

Basic feature description (such as transcript length, GC content, exon number, and transcription level of lncRNA) and comparative analysis (such as Circos, Venn diagram, and boxplot) were completed using TBtools (version 2.088) and Prism (version 8.0.2) [93,94]. To compare the conservation of rice lncRNA, we downloaded the lncRNA sequences of 39 species on CANTATAdb and then used BLAST to perform homologous analysis (version 3.0; E-value < 10^−5^; http://rhesus.amu.edu.pl/CANTATA/index.html, accessed on 29 April 2024) [95]. In addition, the differential expression levels of lncRNA and PCG transcripts were obtained using Cuffdiff [88]. Here, we used|log_2_ (fold change)|values ≥ 0.5, *p*-value ≤ 0.05, and *q* value ≤ 0.05 as thresholds to select significantly differentially expressed lncRNA transcripts (DElncRNAs) and PCG transcripts (DEPCGs). 

### 4.4. Prediction of Trans-Regulated Target PCGs of lncRNAs 

LncRNAs have been found to interact with PCGs in the *trans*-regulation, independent of genomic distance. In consideration of the lncRNAs and PCGs transcripts expression matrix, we applied a weighted gene co-expression network analysis (WGCNA) to establish the co-expression network of rice shoots and roots [96]. To filter out noisy transcripts, all features with a count of less than one (FPKM) in more than 90% of the samples were removed (filter method = MAD; reserved genes = 10,000). Employing step-by-step network construction and module detection, we set the following parameters: the R2 cut-off was 0.85; the power recommended was 16; the scale R2 was 0.83; the minModuleSize was 50; the select maxBlockSize was 5000; the cutHeight was 0.25; the KME cut-off was 0.8. Detailed scale-free network construction information and related data are shown in Figure S1. We scrutinized the relationships between different modules and samples, and the GO (RGAP, http://rice.uga.edu/, accessed on 16 November 2023) enrichment analysis was conducted on the modules of interest to understand the corresponding biological significance [97]. GS and kME values (cutoff of absolute value = 0.5) were combined to find Hub-lncRNAs and Hub-PCGs, and Cytoscape (version 3.9.1) was used to visualize the lncRNA *trans*-regulation co-expression network [98]. 

### 4.5. Prediction of cis-Regulated Target Genes of DElncRNAs 

We could predict the function of lncRNAs by exploring the nearby PCGs of lncRNA in *cis*-regulation. PCGs localized within 100 kb upstream and downstream of the lncRNA loci were screened using Bedtools (version 2.30.0; https://github.com/arq5x/bedtools2/releases, accessed on 16 November 2023). The Pearson correlation coefficient (PCC) between the lncRNAs and PCGs was calculated, and the transcript pairs met the requirements (PCC ≥ 0.9, *p* < 0.05) and were regarded as potential *cis*-regulated target genes of DElncRNAs [99,100]. To speculate on biological functions, GO analysis of potential target PCGs of lncRNAs was similar to the method mentioned above. The relative number of PCGs was visualized using “Word Clouds” (http://www.yyyweb.com/demo/inner-show/word-itout.html, accessed on 21 April 2024).

### 4.6. Identification of Transcription Factors

It has been demonstrated that plant transcription factors play pivotal roles in regulating plant development, metabolic processes, and response to biotic and abiotic stresses. PlantTFDB (version 4.0; https://planttfdb.gao-lab.org/, accessed on 20 November 2023) was utilized to conduct a transcription factors survey for the *cis*-regulated target genes of DElncRNAs [101].

### 4.7. Phylogenetic and Motif Structural Analysis

To understand the evolution of lncRNAs, the “One Step Build an ML Tree” model in TBtools was utilized to conduct the Maximal Likelihood tree (ML-tree). Muscle, trimAI, and IQ-tree were used in combination to perform multiple sequence alignment and phylogenetic tree construction (Bootstrap = 5000). To observe the conserved sequence architecture of the lncRNAs, we predicted the motifs using MEME (version 5.5.5; https://meme-suite.org/meme/tools/meme, accessed on 29 April 2024) [87]. In addition, an Integrative Genomics Viewer (IGV) was operated to perform a visual analysis of lncRNA gene structural and transcriptomic reads [102]. 

## 5. Conclusions

Using transcriptomics and bioinformatics methods, we conducted lncRNA identification and characterized the expression patterns in the seedling roots and shoots of WT and *ososca1.1* under hyperosmolarity and salt stress. Our result compiled lncRNAs into the regulatory pathways of *OSCA* family members for the first time. Furthermore, we pointed out that lncRNAs in roots were more sensitive to hyperosmolarity stress than salt stress compared to PCGs. OsOSCA1.1-related hyperosmolarity stress-responsive lncRNAs enriched in roots and their potential *cis*-regulated genes, which were closely related to transcriptional regulation and signaling transduction, offered potential candidates for future investigations. Numerous studies have shown lncRNAs are involved in regulating plant salt and hyperosmolarity stress responses, while their specific functions are rarely reported. The mechanisms of stress resistance between different crop species or different varieties of the same species may be partially similar. Homozygous T-DNA insertion mutants or CRISPR-Cas9 mutants in our identified lncRNAs could be generated in subsequent research to demonstrate the mechanism of action for lncRNAs. Searching for key genes and applying them in molecular design breeding could help cultivate salt and hyperosmolarity-resistant crop varieties. The *Oryza*-specific *OSlncRNA* gene might provide a paradigm for understanding the important role of non-coding genes in rice’s adaptation to abiotic stress. Our studies help to decode the complex molecular networks involved in plant sensing and adaptation to stressful environments. 

## Data Availability

The original contributions presented in the study are included in the article/Supplementary Materials, and further inquiries can be directed to the corresponding author/s.

## Supplementary Materials

The following supporting information can be downloaded at: https://www.mdpi.com/article/10.3390/ijms25116226/s1.
