# Supplementary material for: Long Noncoding RNAs in Response to Hyperosmolarity Stress, but Not Salt Stress, Were Mainly Enriched in the Rice Roots
Source: Int J Mol Sci. 2024 Jun 5;25(11):6226. doi: 10.3390/ijms25116226 (PMC11172603; doi:10.3390/ijms25116226)
Supplement: Supplementary file 1 [file ijms-25-06226-s001.zip › Supplementary Figures and Tables captions.pdf]

Supplementary Tables

Table S1. Transcriptome-wide information for 2,937 lncRNA in the rice seedlings.

Table S2. Homologous lncRNAs in the rice seedlings with 39 species.

Table S3. Expression of 2,937 lncRNAs in the rice seedlings.

Table S4. lncRNAs and PCGs in 12 different samples.

Table S5. *Trans*-regulated lncRNAs and PCGs in the "Blue" model and "Yellow" model.

Table S6. DEPCGs.

Table S7. DElncRNAs.

Table S8. OsOSCA1.1-related hyperosmolarity stress-responsive lncRNAs and their *cis*-regulated target PCGs in the roots.

Table S9. Summary of TCONS_00017205 conversation in *Oryza*.

Supplementary Figures

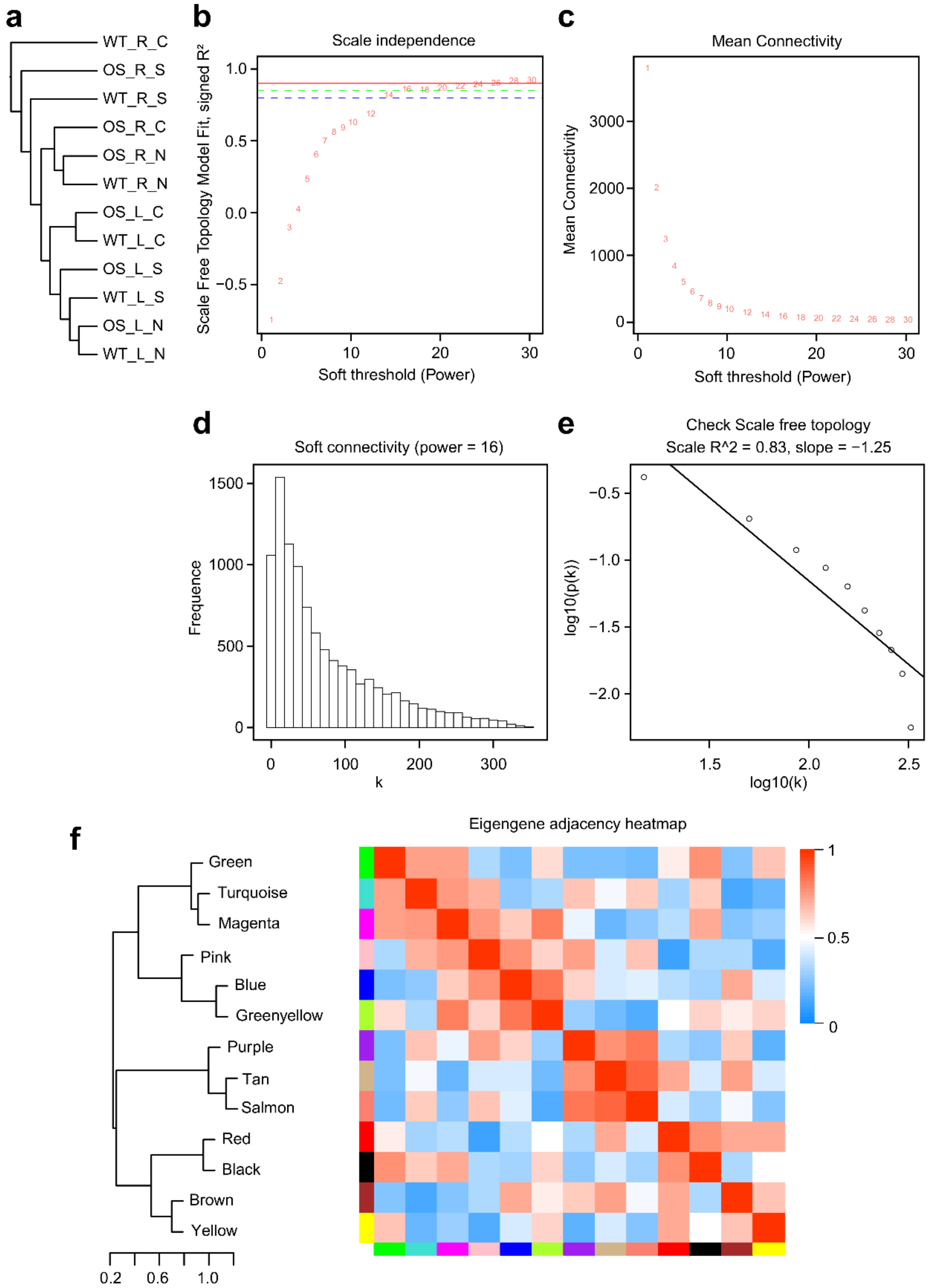

Supplementary Figure S1. Cluster dendrograms and modules in WGCNA. (**a**) Sample cluster of different samples about rice seedlings. No outlier sample group was found. (**b**) Scale

independence state (power recommended = 16). (**c**) Mean connectivity and soft threshold. (**d**) Soft connectivity about frequency and k value. (**e**) Check scale-free topology (scale R2 = 0.83, slope= −1.25). (**f**) Eigengene adjacency heatmap.

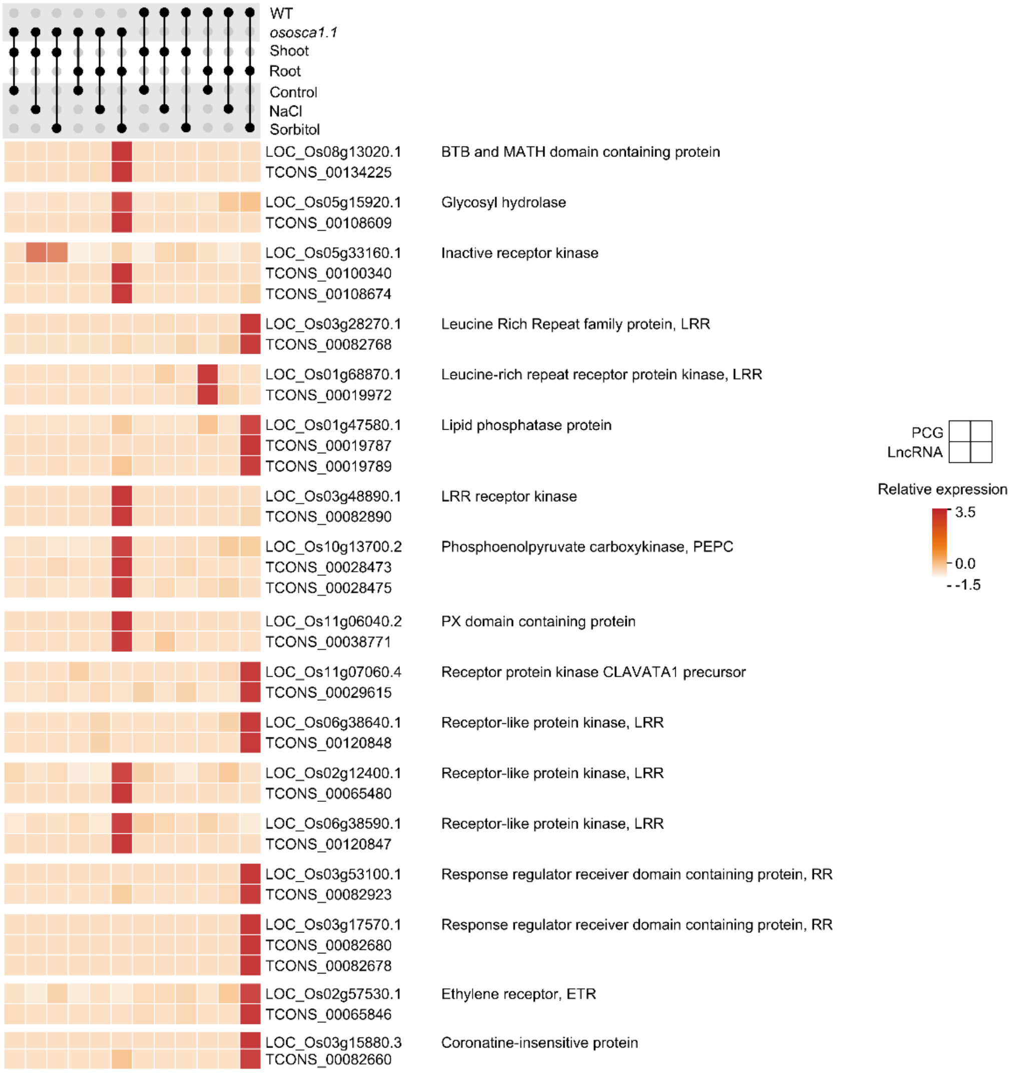

Supplementary Figure S2. Expression heatmaps of potential *cis*-regulated targets of lncRNAs.

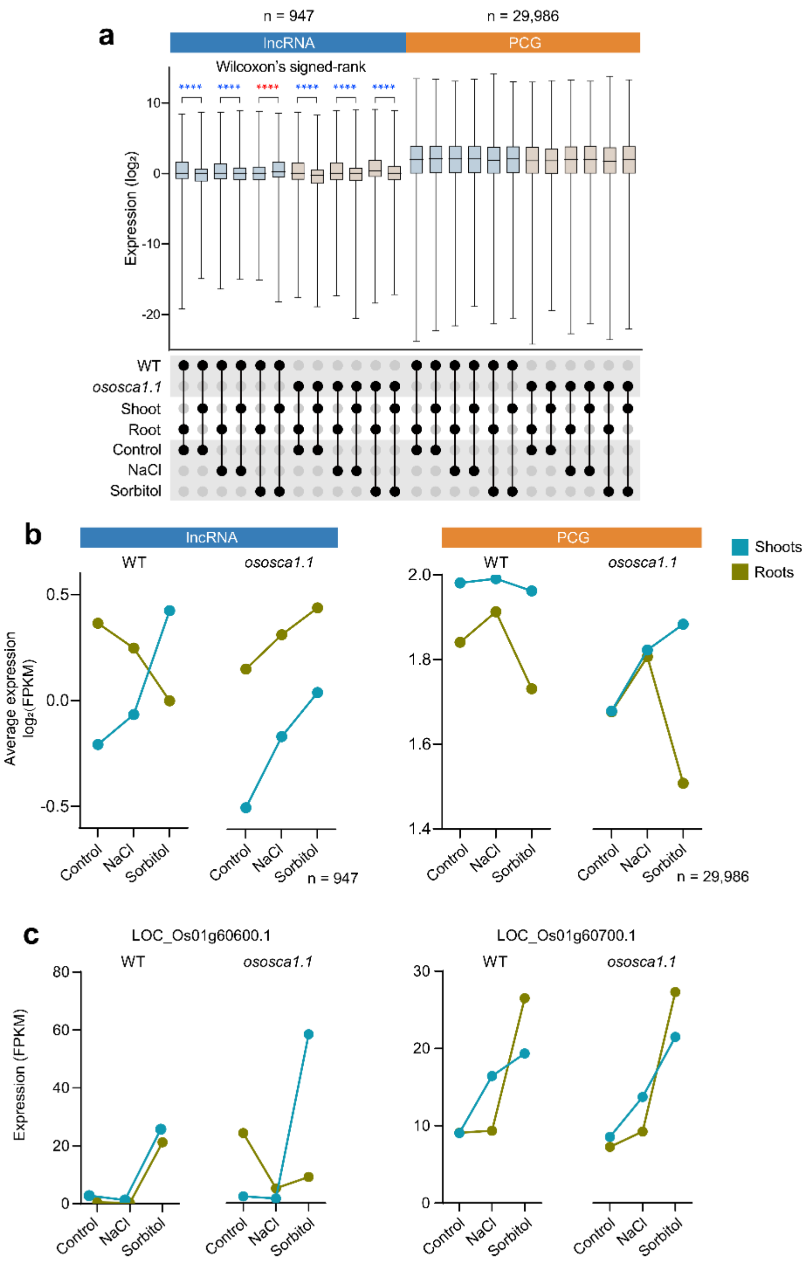

Supplementary Figure S3. Comparison of transcript levels in aboveground and underground parts of rice seedlings. (**a**) Box plots of expression patterns about lncRNAs (n = 947) and PCGs (n = 29,986) in rice roots and shoots (in both WT and *ososca1.1*). (**b**) Comparison of average expression levels about lncRNAs and PCGs in rice roots and shoots. (**c**) Expression patterns of LOC_Os01g60600.1 and LOC_Os01g60700.1 in aboveground and underground parts under different stress treatments.
